# Hydrogen-bonding landscape of the carbamoyl­cyano­nitro­somethanide anion in the crystal structure of its ammonium salt

**DOI:** 10.1107/S2056989021010239

**Published:** 2021-10-13

**Authors:** Kostiantyn V. Domasevitch, Ganna A. Senchyk, Andrey B. Lysenko, Eduard B. Rusanov

**Affiliations:** a Inorganic Chemistry Department, National Taras Shevchenko University of Kyiv, Volodymyrska Str. 64/13, 01601 Kyiv, Ukraine; b Institute of Organic Chemistry, Murmanskaya Str. 4, Kyiv 253660, Ukraine

**Keywords:** crystal structure, carbamoyl­cyano­nitro­somethanide, supra­molecular synthon, hydrogen bonding

## Abstract

The structure of the title salt, ammonium carbamoyl­cyano­nitro­somethanide, NH_4_^+^·C_3_H_2_N_3_O_2_^−^, features the co-existence of different hydrogen-bonding patterns, which are specific to each of the three functional groups (nitroso, carbamoyl and cyano) of the methanide anion. The nitroso O-atoms accept as many as three N—H⋯O bonds from the ammonium cations [N⋯O = 2.688 (3)–3.000 (3) Å] to form chains of fused rhombs [(NH_4_)(O)_2_]. The most prominent bonds of the carbamoyl groups are mutual and they yield 2_1_ helices [N⋯O = 2.903 (2) Å], whereas the cyano N-atoms accept hydrogen bonds from sterically less accessible carbamoyl H-atoms [N⋯N = 3.004 (3) Å]. Two weaker NH_4_^+^⋯O=C bonds [N⋯O = 3.021 (2), 3.017 (2) Å] complete the hydrogen-bonded environment of the carbamoyl groups. A Hirshfeld surface analysis indicates that the most important inter­actions are overwhelmingly O⋯H/H⋯O and N⋯H/H⋯N, in total accounting for 64.1% of the contacts for the individual anions. The relatively simple scheme of these inter­actions allows the delineation of the supra­molecular synthons, which may be applicable to crystal engineering of hydrogen-bonded solids containing polyfunctional methanide anions.

## Abstract 2

The structure of the title salt, ammonium carbamoyl­cyano­nitro­somethanide, NH_4_^+^·C_3_H_2_N_3_O_2_^−^, features the co-existence of different hydrogen-bonding patterns, which are specific to each of the three functional groups (nitroso, carbamoyl and cyano) of the methanide anion. The relatively simple scheme of these inter­actions allows the delineation of the supra­molecular synthons, which may be applicable to crystal engineering of hydrogen-bonded solids containing polyfunctional methanide anions.

## Chemical context

Resonance-stabilized methanide-type anions are excellent ligands in metal–organic chemistry, which reveal a variety of coordination modes toward metal ions (Gerasimchuk, 2019 ▸; Turner *et al.*, 2011 ▸). The rich mol­ecular functionality of such species, as is exemplified by different nitrile-, nitroso- and carbamoyl-substituted derivatives, also predetermines their special properties as potent acceptors of conventional hydrogen bonds. These kinds of inter­actions are important for the solvation and solvatochromism of cyano­anions (Gerasimchuk *et al.*, 2010 ▸) and inter­molecular bonding in the crystal structures of metal complexes (Gerasimchuk *et al.*, 2015 ▸), but it could also influence the specific targeting of cyano­anions in biomedical systems (Gerasimchuk *et al.*, 2007 ▸) and their behavior as anionic components for ionic liquids (Janikowski *et al.*, 2013 ▸). It is worth noting that extensive conjugation and charge delocalization within the mol­ecular frameworks support higher electron densities at all three functional sites (Chesman *et al.*, 2014 ▸), which is beneficial for stronger and more directional inter­actions. Therefore, methanide-type anions are well suited for the crystal engineering of hydrogen-bonded solids with cationic H-atom donors (Turner *et al.*, 2009 ▸). 
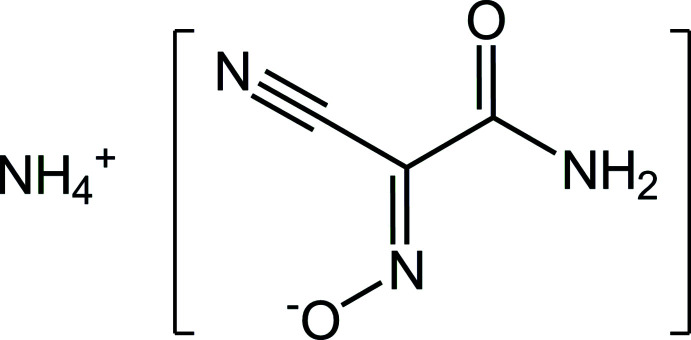


The specific hydrogen-bonding preferences associated with each of the different functional groups at the methanide core could result in a variety of predictable patterns, as well as providing a degree of selectivity for the inter­actions with hydrogen-bond donors. In this view, structurally similar methanides possess a distinct potential for crystal design. For example, either nitroso or carbamoyl groups equally well complement the cyano groups in methanide systems, but the chemical outputs of such functionalization, represented by closely related [ONC(CN)_2_]^−^ and [C(CN)_2_(CONH_2_)]^−^ anions, are rather different with regard to their hydrogen-bonding behavior. The nitroso groups favor direct inter­actions with hydrogen-bond-donor cations and the assembly of cation/anion pairs (Arulsamy *et al.*, 1999 ▸), while the crystal chemistry of carbamoyldi­cyano­methanide is dominated by mutual amide/amide and amide/cyano inter­actions with the generation of less-common anion–anion networks (Turner & Batten, 2010 ▸). The particular combination of nitrile, nitroso and carbamoyl groups in carbamoyl­cyano­nitro­somethanide [ONC(CN)(CONH_2_)]^−^, which is a well known product of the nucleophylic addition of water to [ONC(CN)_2_]^−^ (Arulsamy & Bohle, 2000 ▸), presumably allows one to unite the individual structural trends for the two kinds of anions. One can anti­cipate the assembly of such hybrid hydrogen-bonded structures in a predictable fashion, while taking into account the hierarchy of homo- and heterosynthons formed by each of the functional groups and appropriate hydrogen-bond donors.

In the present contribution, we report the construction of a three-dimensional hydrogen-bonded framework in ammonium carbamoyl­cyano­nitro­somethanide NH_4_(nccm), which features the co-existence and inter­play of the above-mentioned anion–cation and mutual anion–anion inter­actions.

## Structural commentary

**Figure 1:**
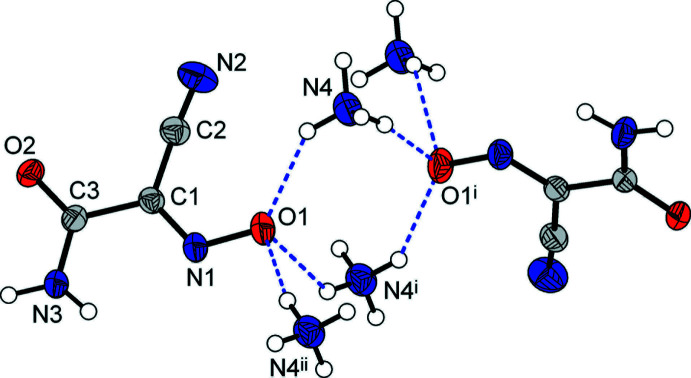
The mol­ecular structure of the title compound with displacement ellipsoids drawn at the 50% probability level. Dotted blue lines indicate N—H⋯O hydrogen bonds [symmetry codes: (i) −*x*, −*y*, *z* − 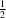 ; (ii) −*x*, −*y*, *z* + 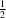 ].

The mol­ecular structure of the title compound is shown in Fig. 1 ▸. This salt is isomorphous with the previously examined Cs analog (Domashevskaya *et al.*, 1989 ▸), which is slightly unusual when considering the very different nature and ionic radii of the cations.

The main geometries of the (nccm)^−^ (or C_3_H_2_N_3_O_2_^−^) anion reveal a highly conjugated structure. The nitroso­cyano­methanide O1/N1/C1/C2/N2 fragment itself is planar within 0.004 Å, being almost coplanar also with the C3/N3/O2 amide fragment [dihedral angle = 3.93 (14)°]. The nitroso group adopts a *trans–anti* configuration with respect to the carbamoyl C=O group, which is the most favorable either for neutral or anionic ONC(CN)—CO*R* species (Ponomareva *et al.*, 1997 ▸; Ponomarova & Domasevitch, 2012 ▸). When compared with the parameters for neutral H(nccm) (Arulsamy & Bohle, 2000 ▸), the deprotonation results in a perceptible lengthening of the double bonds. For example, the carbonyl O2—C3 bond in the title compound is 1.252 (2) Å *versus* 1.228 (3) Å for H(nccm), but the same elongation is relevant also to the N1—C1 bond [1.303 (2) Å], which is significantly longer than in the latter case [1.275 (3) Å].

This is accompanied by a shortening of the N1—O1 bonds, which are particularly sensitive to the protolytic effects. These effects can be precisely traced by gradual shortening of the nitroso bonds for the series H(nccm) [1.356 (2) Å; Arulsamy & Bohle, 2000 ▸] > H(nccm)_2_^−^ in the Rb(18-crown-6)^+^ salt [1.322 (3) Å; Domasevitch *et al.*, 1998 ▸] > (nccm)^−^ in the title salt [1.3117 (19) Å] > (nccm)^−^ in the NMe_4_^+^ salt [1.293 (2) Å; Izgorodina *et al.*, 2010 ▸], in line with the strength of the N—O⋯H bonding. Thus, with relatively strong multiple hydrogen bonds sustained by the nitroso O atoms, the N—O bond order in the title compound is still greater than for the symmetrical hydrogen dioximate anion H(nccm)_2_^−^ [which is structurally similar to more common hydrogen carboxyl­ates (Speakman, 1972 ▸)], but is lower than in NMe_4_(nccm) (one N—H⋯O bond) and also Cs(nccm) [1.297 (8) Å; Domashevskaya *et al.*, 1989 ▸] showing only distal ion–dipole inter­actions of the nitroso group. Such an evolution is clearly reflected in the positions of the ν(NO) bands in the IR spectra (cm^−1^): they are 1098 for H(nccm); 1140 for H(nccm)_2_^−^; 1212 for the title compound; 1253 for NMe_4_(nccm) and 1290 for Cs(nccm), demonstrating the systematic blue shift as the N—O bond order increases.

## Supra­molecular features

Beyond Coulombic attraction forces, the primary kinds of inter­actions for the assembly of the present three-dimensional framework are relatively strong and directional N—H⋯O and N—H⋯N hydrogen bonds (Table 1 ▸). In spite of the high number of hydrogen-bond donors and their multiple inter­actions with a set of closely separated acceptors of different nature, this directional and well-defined bonding facilitates the identification of supra­molecular synthons. This is reminiscent of the behavior of the methanide analogs in NH_4_[C(CN)_2_(CONH_2_)] and NH_4_[ONC(CN)_2_] (Arulsamy *et al.*, 1999 ▸), but is contrary to the structures of comparable nitro­somalono­amides. For example, ammonium violurate exhibits rather weak and bifurcated hydrogen bonding (Nichol & Clegg, 2007 ▸). Also, the cationic ammine in the salt [Ag(NH_3_)_2_](nccm) (Gerasimchuk *et al.*, 2010 ▸) supports only a few weaker and less directional hydrogen bonds.

**Figure 2:**
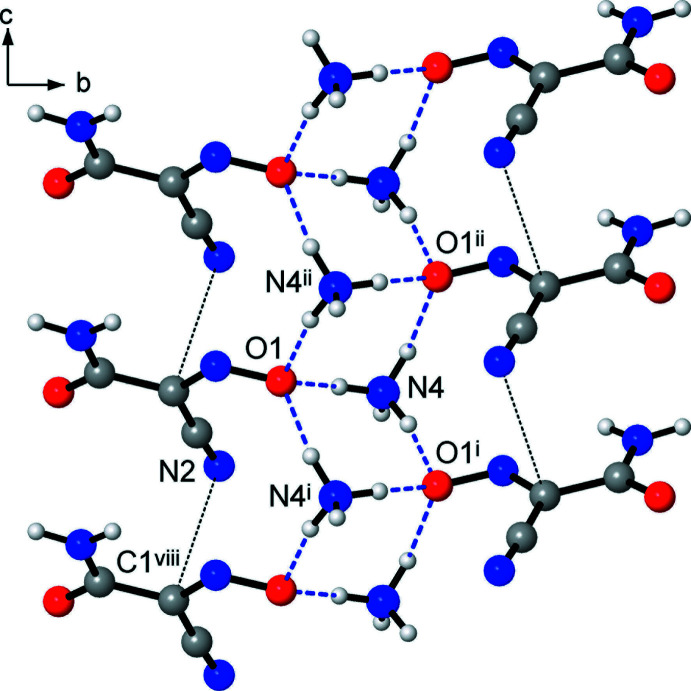
Fragment of the crystal structure showing chains, propagating down the *c*-axis direction, of ammonium/nitroso rhombs sharing opposite edges [symmetry codes: (i) −*x*, −*y*, *z* − 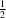 ; (ii) −*x*, −*y*, *z* + 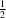 ; (viii) *x*, *y*, *z* − 1].

Both types of O atoms, *i.e*., the nitroso (O1) and carbamoyl (O2) groups, accept three N—H⋯O bonds. However, their bonding preferences are markedly different. All the bonds with the O1 acceptor are sustained with the NH_4_^+^ cations (Fig. 1 ▸), but the principal inter­actions with O2 correspond to the mutual amide/amide type. They represent the strongest bond accepted by O2 [N3⋯O2^v^ = 2.903 (2) Å; N3—H1⋯O^v^ = 161 (2)°; symmetry code (v) −*x*, −*y* + 1, *z* + 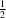 ], as compared with two weaker bonds arising from the distal NH_4_^+^ cations [N4⋯O2 = 3.017 (2), 3.021 (2) A, Fig. 2 ▸, Table 1 ▸].

An important result from the multiple NH_4_^+^⋯ON inter­actions is the assembly of infinite chains running along the *c*-axis direction in the crystal, with the [(NH_4_)_2_(O)_2_] rhombs sharing their opposite edges (Fig. 2 ▸). Two such N—H⋯O bonds are relatively strong [N⋯O = 2.688 (3) and 2.848 (2) Å, Table 1 ▸], whereas N4⋯O1^ii^ [3.000 (3) Å, symmetry code (ii) −*x*, −*y*, *x* + 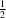 ] exists as a branch of a weaker bifurcated N4—H5⋯(O1,O2) inter­action with the nitroso and carbamoyl acceptors. The present motif is noticeably different from the bonding of NH_4_^+^ cations and nitro­sodicyamomethanide, with the ionic pairs assembled *via* both the O and N atoms of the nitroso groups and only two N—H⋯O inter­actions retained at N⋯O distances of 2.822 (2), 2.881 (2) Å, which are comparable to the two strongest bonds in the title salt (Arulsamy *et al.*, 1999 ▸). Such a discrimination of the nitroso N atom in (nccm)^−^ may be attributed to its lower accessibility, which is in line with the higher steric demands of the carbamoyl group. At the same time, one of the carbamoyl H atoms (which is *trans*-positioned to the C=O bond) is also less accessible and it selectively maintains weaker N—H⋯N bonding to the nitrile acceptor [N3⋯N2^vi^ = 3.004 (3) Å; symmetry code (vi) *x* − 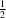 , −*y* + 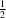 , *z* + 1], very similar to the structure of parent H(nccm) (Arulsamy & Bohle, 2000 ▸).

**Figure 3:**
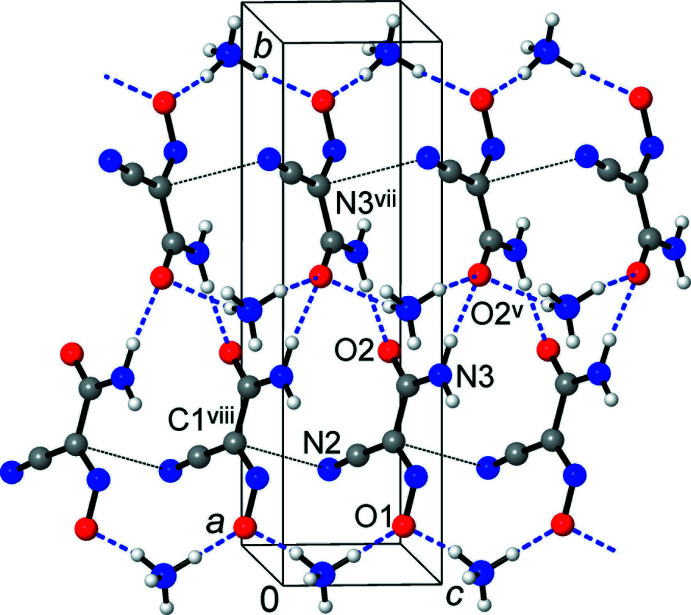
Mutual bonding of CONH_2_ groups, which yields 2_1_ helices propagating along the *c*-axis direction. Stacking inter­actions [*e.g.* N2⋯C1^viii^] are indicated with thin lines [symmetry codes: (v) −*x*, −*y* + 1, *z* + 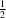 ; (vii) −*x*, −*y* + 1, − 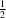 + *z*; (viii) *x*, *y*, *z* − 1].

**Figure 5:**
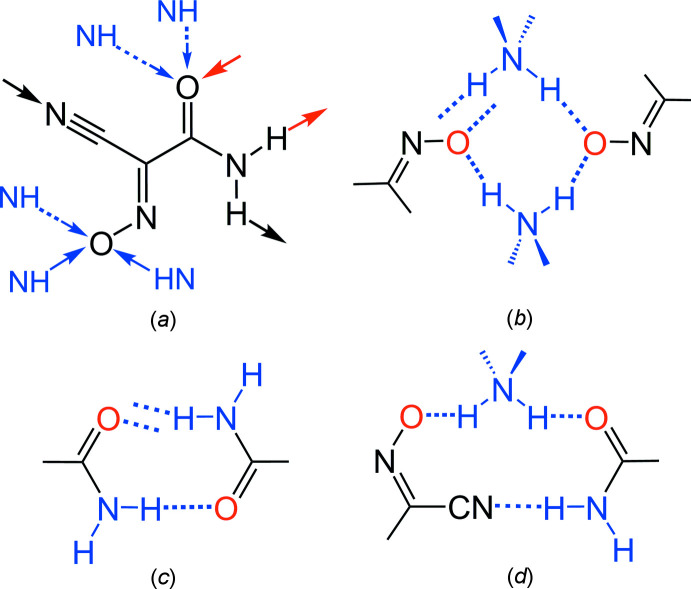
The hydrogen-bonding capacity of the (nccm)^−^ anion. (*a*) Two kinds of mutual inter­actions marked in black and red and bonding with NH_4_^+^ cations marked in blue; (*b*–*d*) three types of supra­molecular synthons identified for the the title compound taking into account a set of strongest inter­actions: ammonium/nitroso chain (*b*), amide/amide chains (*c*) and mutual amide/nitrile bonding (*d*).

One can suppose that the incorporation of tetra­hedral NH_4_^+^ donors itself favors the generation of three-dimensional structures. This is reflected by the formation of one-dimensional helicate motifs as a result of the mutual bonding of the carbamoyl groups (Figs. 3 ▸ and 4 ▸), instead of the more common amide dimers (McMahon *et al.*, 2005 ▸) seen in the NMe_4_^+^ salt (Izgorodina *et al.*, 2010 ▸) and metal complexes of (nccm)^−^ (Domasevitch *et al.*, 1996 ▸). As well, because of the abundance of hydrogen-bond donors, the nitroso O atoms accept auxillary weaker bonds [*i.e.*, N4⋯O1^ii^ = 3.000 (3) Å], which deliver an extension of the anti­cipated discrete pattern based upon single rhombs of [(NH_4_)_2_(O)_2_]. In this view, the hydrogen-bonding preferences of the (nccm)^−^ anion in the title compound could also be applicable to a series of substituted ammonium salts. With fewer N—H donors [NH_4_^+^ > *R*NH_3_^+^ > *R*_2_NH_2_^+^], the possible thinning of the hydrogen-bond shell may result in the elimination of the weakest of the present inter­actions, such as both NH_4_^+^⋯O2 bonds and one of the NH_4_^+^⋯O1 bonds. Therefore, three kinds of supra­molecular synthons, in the form of centrosymmetric amide/amide and ammonium/nitroso dimers as well as the nitrile/amide bonding may be particularly prevalent for crystal engineering with the (nccm)^−^ anion (Fig. 5 ▸).

The columnar packing of (nccm)^−^ anions yields slipped stacks down the *c*-axis direction, with an inter­planar distance of 3.32 Å (Figs. 2 ▸ and 3 ▸). This feature is similar to the structures of cyano­methanide species examined by Chesman *et al.* (2014 ▸), which typically support stacks at 3.15–3.30 Å. However, the overlaps of the (nccm)^−^ skeletons are minor [as indicated by a large slippage angle of 54.9 (2)°] and actually only the nitrile fragment is involved in the stacking with the methanide fragment. The shortest contact between translation-related anions is N2⋯C1^viii^ = 3.357 (2) Å [symmetry code: (viii) *x*, *y*, *z* – 1]. This stacking is less significant for (nccm)^−^ salts due to the prevalent role of hydrogen bonding, which is a primary anion–anion inter­action for carbamoyl-substituted methanides (Chesman *et al.*, 2014 ▸).

## Hirshfeld analysis

**Figure 6:**
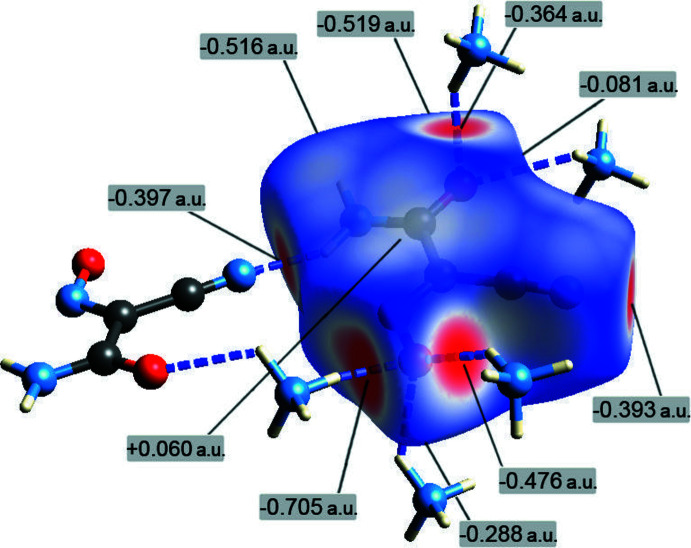
The Hirshfeld surface of the (nccm)^−^ anion mapped over *d*_norm_ in the color range −0.71 (red) to 1.05 a.u. (blue), in the environment of the hydrogen-bonded ammonium cations and one (out of three) (nccm)^−^ anion.

The supra­molecular inter­actions in the title structure were further investigated by Hirshfeld surface analysis (Spackman & Byrom, 1997 ▸; McKinnon *et al.*, 2004 ▸; Hirshfeld, 1977 ▸; Spackman & McKinnon, 2002 ▸) performed with *CrystalExplorer17* (Turner *et al.*, 2017 ▸). The Hirshfeld surface of the individual (nccm)^−^ anion mapped over *d*_norm_, using a fixed color scale of −0.71 (red) to 1.05 a.u. (blue), reveals a set of red spots associated with the inter­action sites (Fig. 6 ▸). The most intense spot (−0.708 a.u.) reflects the very short NH_4_^+^-O-nitroso bond, whereas a group of six almost equally prominent spots (−0.393 to −0.519 a.u.) correspond to the mutual amide/amide, amide/nitrile, one NH_4_^+^-O-nitroso and one NH_4_^+^-O-carbamoyl bonds. A third spot in the region of the nitroso-O acceptor is less intense (−0.288 a.u.), while the additional NH_4_^+^—O-carbamoyl bond has only a minor indication of −0.081 a.u.

**Figure 7:**
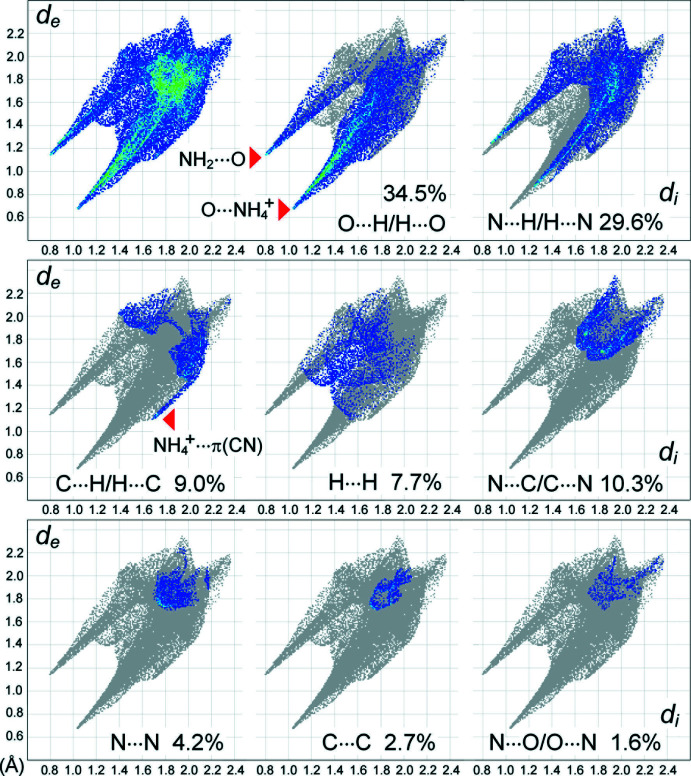
Two-dimensional fingerprint plots for the anions of the title compound, and delineated into the principal contributions of O⋯H/H⋯O, N⋯H/H⋯N, C⋯H/H⋯C, H⋯H, N⋯C/C⋯N, N⋯N, C⋯C and N⋯O/O⋯N contacts. Other minor contributors are C⋯O/O⋯C contacts (0.3%).

The two-dimensional fingerprint plots (Fig. 7 ▸) are consistent with the prevalence of hydrogen bonding in the structure. For the individual NH_4_^+^ cations, as much as 57.3% of their surface are H⋯O contacts. The H⋯N contacts account for only 20.1% (H⋯H and H⋯C are 20.1% and 2.5%, respectively), which suggests a rather high selectivity in the bonding of NH_4_^+^ cations to the O-acceptor sites. The plots for the anion are even more informative. The short separations are overwhelmingly hydrogen-bond contacts, accounting for 64.1% of the surface. The O⋯H/H⋯O fraction of 34.5% appears on the plot as a pair of sharp spikes pointing to the lower left, with the upper spike representing entirely H⋯O of the amide/amide synthon (the shortest contact is 2.0 Å), while the more intense and longer lower spike is due to a reciprocal O⋯H bond superimposed with points from stronger and more numerous O⋯H (NH_4_^+^) contacts (the shortest is 1.7 Å). In the case of N⋯H/H⋯N type (29.6%), two spikes are shorter (2.2 Å) and nearly symmetrical, indicating the mutual character of this weaker bonding. Although the C⋯H/H⋯C contacts of the anion (9.0%) are mostly mutual, the plot also features a small but relatively sharp spike from C⋯NH_4_^+^ contacts (2.8 Å), which has a complementary donor part at the plot for individual NH_4_^+^ cations (not shown here). This very distal inter­action may be rationalized as an NH⋯π(C≡N) bond, with the distances N4⋯*Cg*(C2≡N2) = 3.584 (3); H⋯*Cg*(C2≡N2) = 2.89 (3) Å and N4H⋯*Cg*(C2≡N2) = 136 (3)° (*Cg* is the mid-point of the C2—N2 bond). A similar contact was observed for NH_4_{ONC(CN)_2_} (Arulsamy *et al.*, 1999 ▸). Stacking inter­actions in the title compound are also important. They contribute in total 18.8% of the contacts represented by the N⋯C/C⋯N, N⋯N, C⋯C and N⋯O/O⋯N types, all of which have a very similar nature and metrics (the shortest is N⋯C = 3.3 Å). In summary, the results of Hirshfeld surface analysis effectively illustrate the predominant roles of multiple ammonium/nitroso, mutual amide/amide and amide-nitrile inter­actions as the main supra­molecular synthons.

## Synthesis and crystallization

The 2-cyano-2-iso­nitro­soacetamide H(nccm) was prepared by nitro­sation of cyano­acetamide (Gerasimchuk *et al.*, 2010 ▸). It is a relatively weak acid (p*K*_α_ = 5.03; Klaus *et al.*, 2015 ▸) and therefore the compound NH_4_(nccm) is unstable, readily losing ammonia in air within a period of several days. When slowly evaporated, its aqueous or methano­lic solutions lose ammonia first and then H(nccm) crystallizes.

For the preparation of the title compound, 0.339 g of H(nccm) (3 mmol) was dissolved in 10 ml of methanol at 303–313 K and 0.6 ml of 25% aqueous ammonia (8 mmol) were added to form a clear pale-yellow solution. It was placed, in an open vial, inside the larger stoppered flask containing mixture of 50 ml of 2-propanol and 1 ml of 25% aqueous ammonia. Slow inter­diffusion of the solvents through the gaseous phase resulted in the precipitation of large pale-yellow NH_4_(nccm) crystals over a period of 30 d. The yield was 0.250 g (64%). Analysis (%) calculated for C_3_H_6_N_4_O_2_: C 27.69, H 4.65, N 43.07; found: C 28.01, H 4. 85, N 42.68. IR (KBr, cm^−1^): 500 *w*, 668 *s*, 766 *m*, 1022 *s*, 1092 *s*, 1144 *s*, 1172 *s*, 1212 *s*, 1402 *s*, 1600 *s*, 1686 *vs*, 2218 *m*, 3170 *br*, 3302 *br*, 3450 *s*.

## Refinement

Crystal data, data collection and structure refinement details are summarized in Table 2 ▸. All hydrogen atoms were located and then refined isotropically. Soft similarity restraints were applied to four N—H bond lengths and six H—N—H bond angles of the ammonium cations.

## Supplementary Material

Crystal structure: contains datablock(s) global, I. DOI: 10.1107/S2056989021010239/hb7989sup1.cif

Structure factors: contains datablock(s) I. DOI: 10.1107/S2056989021010239/hb7989Isup2.hkl

Supporting information file. DOI: 10.1107/S2056989021010239/hb7989Isup3.cml

CCDC reference: 2113575

Additional supporting information:  crystallographic information; 3D view; checkCIF report

## Figures and Tables

**Figure 4:**
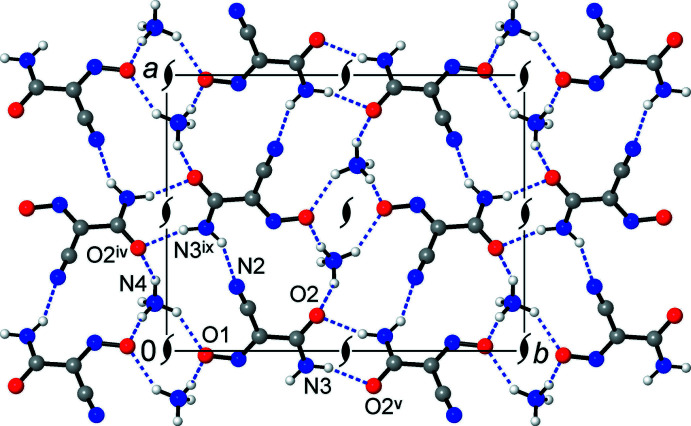
Structure of the title compound, viewed in a projection onto the *ab* plane, showing the co-existence and inter­play of the three main supra­molecular motifs in the form of ammonium/nitroso chains, amide/amide chains (both of which are situated across 2_1_ axes and are orthogonal to the drawing plane) and amide–nitrile mutual bonding [symmetry codes: (iv) −*x* + 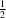 , *y* − 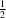 , *z* − 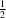 ; (v) −*x*, −*y* + 1, *z* + 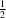 ; (ix) *x* + 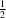 , −*y* + 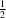 , *z*].

**Table 1:** Hydrogen-bond geometry (Å, °)

*D*—H⋯*A*	*D*—H	H⋯*A*	*D*⋯*A*	*D*—H⋯*A*
N4—H3⋯O1	0.89 (2)	2.07 (2)	2.848 (2)	145 (2)
N4—H4⋯O1^i^	0.92 (2)	1.78 (2)	2.688 (3)	167 (2)
N4—H5⋯O1^ii^	0.89 (2)	2.35 (3)	3.000 (3)	129 (3)
N4—H5⋯O2^iii^	0.89 (2)	2.46 (3)	3.021 (2)	122 (3)
N4—H6⋯O2^iv^	0.93 (2)	2.19 (2)	3.017 (2)	148 (2)
N3—H1⋯O2^v^	0.92 (3)	2.02 (3)	2.903 (2)	161 (2)
N3—H2⋯N2^vi^	0.85 (3)	2.24 (3)	3.004 (3)	149 (3)

Symmetry codes: (i) -x, -y, z-{\script{1\over 2}}; (ii) -x, -y, z+{\script{1\over 2}}; (iii) -x+{\script{1\over 2}}, y-{\script{1\over 2}}, z+{\script{1\over 2}}; (iv) -x+{\script{1\over 2}}, y-{\script{1\over 2}}, z-{\script{1\over 2}}; (v) -x, -y+1, z+{\script{1\over 2}}; (vi) x-{\script{1\over 2}}, -y+{\script{1\over 2}}, z+1.

**Table 2:** Experimental details

Crystal data	
Chemical formula	NH_4_^+^·C_3_H_2_N_3_O_2_^−^
*M*_r_	130.12
Crystal system, space group	Orthorhombic, *P* *n* *a*2_1_
Temperature (K)	173
*a*, *b*, *c* (Å)	10.7174 (5), 13.8944 (7), 4.0643 (2)
*V* (Å^3^)	605.22 (5)
*Z*	4
Radiation type	Mo *K*α
μ (mm^−1^)	0.12
Crystal size (mm)	0.37 × 0.30 × 0.21

Data collection	
Diffractometer	Bruker APEXII CCD
No. of measured, independent and observed [*I* > 2σ(*I*)] reflections	7798, 1420, 1304
*R*_int_	0.031
(sin θ/λ)_max_ (Å^−1^)	0.663

Refinement	
*R*[*F* ^2^ > 2σ(*F* ^2^)], *wR*(*F* ^2^), *S*	0.032, 0.081, 1.10
No. of reflections	1420
No. of parameters	106
No. of restraints	22
H-atom treatment	All H-atom parameters refined
Δρ_max_, Δρ_min_ (e Å^−3^)	0.19, −0.14

Computer programs: *SMART-NT* (Bruker, 1998 ▸), *SAINT-NT* (Bruker, 1999 ▸), *SHELXS97* (Sheldrick, 2008 ▸), *SHELXL2014*/7 (Sheldrick, 2015 ▸), *DIAMOND* (Brandenburg, 1999 ▸) and *WinGX* (Farrugia, 2012 ▸).

## supplementary crystallographic information

### Crystal data

**Table:**

NH_4_^+^·C_3_H_2_N_3_O_2_^−^	*D*_x_ = 1.428 Mg m^−^^3^
*M**_r_* = 130.12	Mo *K*α radiation, λ = 0.71073 Å
Orthorhombic, *P**n**a*2_1_	Cell parameters from 2828 reflections
*a* = 10.7174 (5) Å	θ = 2.4–25.3°
*b* = 13.8944 (7) Å	µ = 0.12 mm^−^^1^
*c* = 4.0643 (2) Å	*T* = 173 K
*V* = 605.22 (5) Å^3^	Prism, yellow
*Z* = 4	0.37 × 0.30 × 0.21 mm
*F*(000) = 272	

### Data collection

**Table:**

Bruker APEXII CCD diffractometer	1304 reflections with *I* > 2σ(*I*)
Radiation source: fine-focus sealed tube	*R*_int_ = 0.031
Graphite monochromator	θ_max_ = 28.1°, θ_min_ = 2.4°
φ and ω scans	*h* = −14→14
7798 measured reflections	*k* = −18→18
1420 independent reflections	*l* = −5→5

### Refinement

**Table:**

Refinement on *F*^2^	Primary atom site location: structure-invariant direct methods
Least-squares matrix: full	Secondary atom site location: difference Fourier map
*R*[*F*^2^ > 2σ(*F*^2^)] = 0.032	Hydrogen site location: difference Fourier map
*wR*(*F*^2^) = 0.081	All H-atom parameters refined
*S* = 1.10	*w* = 1/[σ^2^(*F*_o_^2^) + (0.0439*P*)^2^ + 0.0604*P*] where *P* = (*F*_o_^2^ + 2*F*_c_^2^)/3
1420 reflections	(Δ/σ)_max_ < 0.001
106 parameters	Δρ_max_ = 0.19 e Å^−^^3^
22 restraints	Δρ_min_ = −0.14 e Å^−^^3^

### Special details

**Table:**

Geometry. All esds (except the esd in the dihedral angle between two l.s. planes) are estimated using the full covariance matrix. The cell esds are taken into account individually in the estimation of esds in distances, angles and torsion angles; correlations between esds in cell parameters are only used when they are defined by crystal symmetry. An approximate (isotropic) treatment of cell esds is used for estimating esds involving l.s. planes.

### Fractional atomic coordinates and isotropic or equivalent isotropic displacement parameters (Å^2^)

**Table:**

	*x*	*y*	*z*	*U*_iso_*/*U*_eq_	
O1	−0.01669 (13)	0.10947 (9)	0.7570 (4)	0.0365 (4)	
O2	0.12729 (11)	0.42081 (9)	0.7040 (4)	0.0306 (3)	
N1	−0.02966 (14)	0.20089 (10)	0.8299 (5)	0.0285 (4)	
N2	0.23276 (19)	0.19606 (15)	0.3518 (6)	0.0472 (5)	
N3	−0.05305 (16)	0.38965 (12)	0.9758 (5)	0.0319 (4)	
N4	0.17308 (17)	−0.03313 (12)	0.7032 (5)	0.0367 (4)	
C1	0.05333 (15)	0.25999 (12)	0.7110 (5)	0.0242 (4)	
C2	0.15514 (18)	0.22726 (14)	0.5109 (6)	0.0301 (4)	
C3	0.04438 (16)	0.36355 (12)	0.7975 (5)	0.0243 (4)	
H1	−0.057 (2)	0.4525 (18)	1.043 (8)	0.045 (7)*	
H2	−0.104 (3)	0.346 (2)	1.039 (8)	0.054 (8)*	
H3	0.1437 (19)	0.0263 (14)	0.727 (7)	0.046 (7)*	
H4	0.125 (2)	−0.0681 (17)	0.557 (6)	0.052 (8)*	
H5	0.177 (3)	−0.066 (2)	0.891 (7)	0.15 (2)*	
H6	0.251 (2)	−0.0308 (18)	0.605 (7)	0.068 (10)*	

### Atomic displacement parameters (Å^2^)

**Table:**

	*U*^11^	*U*^22^	*U*^33^	*U*^12^	*U*^13^	*U*^23^
O1	0.0414 (8)	0.0202 (6)	0.0479 (11)	−0.0014 (5)	0.0053 (8)	−0.0033 (6)
O2	0.0263 (6)	0.0245 (6)	0.0410 (8)	−0.0038 (5)	0.0026 (6)	0.0051 (6)
N1	0.0318 (8)	0.0209 (7)	0.0329 (9)	−0.0005 (6)	0.0012 (7)	−0.0016 (7)
N2	0.0415 (10)	0.0541 (11)	0.0459 (13)	0.0128 (8)	0.0116 (10)	−0.0012 (9)
N3	0.0315 (8)	0.0215 (8)	0.0427 (11)	−0.0039 (6)	0.0089 (8)	−0.0041 (7)
N4	0.0406 (9)	0.0299 (8)	0.0396 (10)	0.0044 (7)	−0.0045 (10)	−0.0042 (9)
C1	0.0248 (8)	0.0222 (8)	0.0256 (8)	0.0024 (6)	−0.0012 (8)	0.0010 (7)
C2	0.0298 (9)	0.0287 (9)	0.0318 (10)	0.0030 (7)	0.0013 (9)	0.0043 (8)
C3	0.0234 (8)	0.0224 (8)	0.0270 (10)	−0.0008 (6)	−0.0034 (7)	0.0027 (7)

### Geometric parameters (Å, º)

**Table:**

O1—N1	1.3117 (19)	N4—H3	0.89 (2)
O2—C3	1.252 (2)	N4—H4	0.924 (19)
N1—C1	1.303 (2)	N4—H5	0.89 (2)
N2—C2	1.140 (3)	N4—H6	0.93 (2)
N3—C3	1.322 (3)	C1—C2	1.435 (3)
N3—H1	0.92 (3)	C1—C3	1.484 (2)
N3—H2	0.85 (3)		
			
C1—N1—O1	117.00 (15)	H5—N4—H6	110 (2)
C3—N3—H1	117.6 (17)	N1—C1—C2	121.97 (16)
C3—N3—H2	118 (2)	N1—C1—C3	118.61 (16)
H1—N3—H2	124 (3)	C2—C1—C3	119.38 (15)
H3—N4—H4	111.2 (17)	N2—C2—C1	176.0 (2)
H3—N4—H5	114 (2)	O2—C3—N3	123.56 (17)
H4—N4—H5	108 (2)	O2—C3—C1	119.90 (16)
H3—N4—H6	109.6 (18)	N3—C3—C1	116.54 (15)
H4—N4—H6	104.0 (17)		
			
O1—N1—C1—C2	−0.4 (3)	C2—C1—C3—O2	−2.1 (3)
O1—N1—C1—C3	−177.92 (16)	N1—C1—C3—N3	−4.0 (3)
N1—C1—C3—O2	175.4 (2)	C2—C1—C3—N3	178.45 (19)

### Hydrogen-bond geometry (Å, º)

**Table:**

*D*—H···*A*	*D*—H	H···*A*	*D*···*A*	*D*—H···*A*
N4—H3···O1	0.89 (2)	2.07 (2)	2.848 (2)	145 (2)
N4—H4···O1^i^	0.92 (2)	1.78 (2)	2.688 (3)	167 (2)
N4—H5···O1^ii^	0.89 (2)	2.35 (3)	3.000 (3)	129 (3)
N4—H5···O2^iii^	0.89 (2)	2.46 (3)	3.021 (2)	122 (3)
N4—H6···O2^iv^	0.93 (2)	2.19 (2)	3.017 (2)	148 (2)
N3—H1···O2^v^	0.92 (3)	2.02 (3)	2.903 (2)	161 (2)
N3—H2···N2^vi^	0.85 (3)	2.24 (3)	3.004 (3)	149 (3)

Symmetry codes: (i) −*x*, −*y*, *z*−1/2; (ii) −*x*, −*y*, *z*+1/2; (iii) −*x*+1/2, *y*−1/2, *z*+1/2; (iv) −*x*+1/2, *y*−1/2, *z*−1/2; (v) −*x*, −*y*+1, *z*+1/2; (vi) *x*−1/2, −*y*+1/2, *z*+1.

## References

[bb1] Arulsamy, N. & Bohle, D. S. (2000). *J. Org. Chem.* **65**, 1139–1143. 10.1021/jo991614t 10814065

[bb2] Arulsamy, N., Bohle, D. S. & Doletski, B. G. (1999). *Inorg. Chem.* **38**, 2709–2715.

[bb3] Brandenburg, K. (1999). *DIAMOND.* Crystal Impact GbR, Bonn, Germany.

[bb4] Bruker (1998). *SMART-NT*. Bruker AXS Inc., Madison, Wisconsin, USA.

[bb5] Bruker (1999). *SAINT-NT*. Bruker AXS Inc., Madison, Wisconsin, USA.

[bb6] Chesman, A. S. R., Hodgson, J. L., Izgorodina, E. I., Urbatsch, A., Turner, D. R., Deacon, G. B. & Batten, S. R. (2014). *Cryst. Growth Des.* **14**, 1922–1932.

[bb7] Domasevitch, K. V., Ponomareva, V. V., Rusanov, E. B., Gelbrich, T., Sieler, J. & Skopenko, V. V. (1998). *Inorg. Chim. Acta*, **268**, 93–101.

[bb8] Domasevitch, K. V., Skopenko, V. V. & Rusanov, E. B. (1996). *Z. Naturforsch. Teil B*, **51**, 832–837.

[bb9] Domashevskaya, O. A., Mazus, M. D., Gerasimchuk, N. N., Dvorkin, A. A. & Simonov, Yu. A. (1989). *Zh. Neorg. Khimii* **34**, 1656–1660.

[bb10] Farrugia, L. J. (2012). *J. Appl. Cryst.* **45**, 849–854.

[bb11] Gerasimchuk, N. (2019). *Dalton Trans.* **48**, 7985–8013. 10.1039/c9dt01057b 31090771

[bb12] Gerasimchuk, N., Esaulenko, A. N., Dalley, K. N. & Moore, C. (2010). *Dalton Trans.* **39**, 749–764. 10.1039/b915603h 20066220

[bb13] Gerasimchuk, N., Maher, T., Durham, P., Domasevitch, K. V., Wilking, J. & Mokhir, A. (2007). *Inorg. Chem.* **46**, 7268–7284. 10.1021/ic061354f 17676728

[bb14] Gerasimchuk, N. N., Guzei, I. & Sipos, P. (2015). *Curr. Inorg. Chem.* **5**, 38–63.

[bb15] Hirshfeld, F. L. (1977). *Theor. Chim. Acta*, **44**, 129–138.

[bb16] Izgorodina, E. I., Chesman, A. S. R., Turner, D. R., Deacon, G. B. & Batten, S. R. (2010). *J. Phys. Chem. B*, **114**, 16517–16527. 10.1021/jp108550z 21086972

[bb17] Janikowski, J., Razali, M. R., Forsyth, C. M., Nairn, K. M., Batten, S. R., MacFarlane, D. R. & Pringle, J. M. (2013). *ChemPlusChem*, **78**, 486–497.

[bb18] Klaus, D. R., Keene, M., Silchenko, S., Berezin, M. & Gerasimchuk, N. (2015). *Inorg. Chem.* **54**, 1890–1900. 10.1021/ic502805h PMC7441041 25615022

[bb19] McKinnon, J. J., Spackman, M. A. & Mitchell, A. S. (2004). *Acta Cryst.* B**60**, 627–668. 10.1107/S0108768104020300 15534375

[bb20] McMahon, J. A., Bis, J. A., Vishweshwar, P., Shattock, T. R., McLaughlin, O. L. & Zaworotko, M. J. (2005). *Z. Kristallogr.* **220**, 340–350.

[bb21] Nichol, G. S. & Clegg, W. (2007). *Acta Cryst.* C**63**, o609–o612. 10.1107/S0108270107044241 17917236

[bb22] Ponomareva, V. V., Skopenko, V. V., Domasevitch, K. V., Sieler, J. & Gelbrich, T. (1997). *Z. Naturforsch.* **52**, 901–905.

[bb23] Ponomarova, V. V. & Domasevitch, K. V. (2012). *Acta Cryst.* C**68**, o359–o361. 10.1107/S0108270112034208 22935504

[bb24] Sheldrick, G. M. (2008). *Acta Cryst.* A**64**, 112–122. 10.1107/S0108767307043930 18156677

[bb25] Sheldrick, G. M. (2015). *Acta Cryst.* C**71**, 3–8.

[bb26] Spackman, M. A. & Byrom, P. G. A. (1997). *Chem. Phys. Lett.* **267**, 215–220.

[bb27] Spackman, M. A. & McKinnon, J. J. (2002). *CrystEngComm*, **4**, 378–392.

[bb28] Speakman, J. C. (1972). *Structure and Bonding*, Vol. 12, pp. 141–199. Berlin, Heidelberg: Springer.

[bb29] Turner, D. R. & Batten, S. R. (2010). *Cryst. Growth Des.* **10**, 2501–2508.

[bb30] Turner, D. R., Chesman, A. S. R., Murray, K. S., Deacon, G. B. & Batten, S. R. (2011). *Chem. Commun.* **47**, 10189–10210. 10.1039/c1cc11909e 21773626

[bb31] Turner, D. R., MacDonald, R., Lee, W. T. & Batten, S. R. (2009). *CrystEngComm*, **11**, 298–305.

[bb32] Turner, M. J., McKinnon, J. J., Wolff, S. K., Grimwood, D. J., Spackman, P. R., Jayatilaka, D. & Spackman, M. A. (2017). *CrystalExplorer17*. University of Western Australia. http://crystalexplorer.scb.uwa.edu.au/

